# The Biofunctional Effects of Mesima as a Radiosensitizer for Hepatocellular Carcinoma

**DOI:** 10.3390/ijms21030871

**Published:** 2020-01-29

**Authors:** Youn Kyoung Jeong, Ju Yeon Oh, Jae Kuk Yoo, Sun Ha Lim, Eun Ho Kim

**Affiliations:** 1 Radiation Nonclinical Center, Korea Institute of Radiological and Medical Sciences, Seoul 01812, Korea; amy3523@kirams.re.kr; 2 Laboratory of Biochemistry, School of Life Sciences and Biotechnology, Korea University, Seongbuk-gu, Seoul 136-701, Korea; ojo5295@naver.com; 3 Han Kook Shin Yak Pharmaceutical Co., Ltd., Nonsan 33023, Korea; yjk125@daum.net; 4 Department of Biochemistry, School of Medicine, Daegu Catholic University, 33, 17-gil, Duryugongwon-ro, Nam-gu, Daegu 705-718, Korea; sunha112@cu.ac.kr

**Keywords:** mesima, HCC, radiation, radiosensitivity

## Abstract

The tropical basidiomycete fungus *Phellinus linteus* (Mesima) exhibits anti-tumor, anti-angiogenic, and immunomodulatory properties in various cancers including prostate, colon, and lung cancer along with melanoma by, for example, inducing apoptosis or cell cycle arrest. However, whether medina also facilitates treatment of hepatocellular carcinoma (HCC), the third global cause of cancer deaths, remains unknown. Here, we examined its potential as a radiosensitizer in HCC radiotherapy using human HCC Hep3B and HepG2 cell lines and xenograft tumors. Mesima pretreatment significantly enhanced HCC cell radiosensitivity in vitro and the combination of mesima + radiation treatment significantly reduced xenograft tumor growth and size in vivo compared to those with single treatments. Mechanistically, mesima significantly enhanced radiotherapy efficiency by inhibiting tumor cell survival through inducing apoptosis (assessed via annexin V), impairing cell cycle regulation (shown by flow cytometry), and reducing radiation-induced DNA damage repair (measured via γ-H2AX foci). Combination treatment also facilitated autophagic cell death beyond that from single treatments (assessed by quantifying stained acidic vesicular organelles), and diminished tumor cell metastatic potentials (shown by wound and Transwell assays). These findings support the synergistic anti-tumor effects of mesima combined with radiation and suggest scientific evidence for mesima as a radiosensitizer in HCC.

## 1. Introduction

Hepatocellular carcinoma (HCC) is a major contributor to mortality and the third general reason of cancer-related deaths in the world [1,2]. HCC is often detected at an advanced stage or when the patient presents with advanced liver cirrhosis at the time of diagnosis. Recommended treatment choices for HCC may be either curative or palliative, depending on cancer stage. Curative treatments, which accomplish the best results, cover liver resection and transplantation, whereas palliative therapy comprises tumor ablation, embolization, radiotherapy, and chemotherapy [3]. In the field of radiation therapy in particular, technological advances including computed tomography-based planning and stereotactic radiosurgery have been performed for tumors by reducing the normal tissue. However, these physics of radiation do not allow for the complete avoidance of normal tissue, at least in terms of current technology [4]. To clear these problems, one of the promising alternative treatments is the development of radiosensitizer candidates for overcoming radioresistance. Radiosensitizers could selectively enhance killing of cancer cells by regulating cell cycle and microenvionmental properties. To be select as a radiosensitizer, candidates should be highly effective and low-toxicity [5].

Mesima is a substance that enhances the immunological activities containing protein-bounded polysaccharide (PPLA) that is obtained in the process of massively cultivating, extracting, and refining pure mycelium from the fruit body of *Phellinus linteus* that is also called Sang-Hwang (galenical name), a kind of mushroom. *Phellinus linteus* (PL, mesima), a basidiomycete fungus located mostly in tropical America, Africa, and Asia, has been utilized as medicinal mushroom in traditional medicine for treating a large number of human malignancies [4]. Investigations have shown that the water-soluble fraction of mesima is biologically active, with the active ingredient likely constituting a polysaccharide [5,6,7]; in addition, immunostimulatory and anti-tumor activities have also been reported [8,9,10]. Recently, mesima has been found to be effective for blocking the growth of various prostate cancer cell lines by apoptosis and cell cycle blockade, and similarly decreases tumor growth, invasion, and angiogenesis along with altering Wnt/b-catenin in human colon cancer cells [11,12,13]. Moreover, the anticancer effect of mesima has been investigated, as evidenced by blocking of invasive melanoma cells through decreasing mRNA levels of urokinase-plasminogen activator (uPA), and by the suppression of pulmonary metastasis in mice [14]. Mesima also suppresses proliferation by inhibiting cyclin-dependent kinases cdk2, 4, and 6, and inducing cell death through the activation of caspase 3 in lung cancer cells [10]. Such reports of mesima anti-tumor, anti-angiogenic, and immunomodulatory properties have stimulated considerable interest in Asia for its development as an anti-cancer drug. In addition, it has been confirmed that mesima is not harmful for the human body by the clinical toxicity test of the toxicology research center of the Korea Research Institute of Chemical Technology (Good Laboratory Practice (GLP)-approved research institute).

Therefore, the purpose of our study was to define the potential of mesima as a radiosensitizer in HCC radiotherapy, to identify the best combination strategies to potentiate the anticancer effect on HCC, and to examine the possible mechanisms of action. 

## 2. Results

### 2.1. Mesima Sensitized HCC to Radiation In Vitro and In Vivo

**Figure 1:**
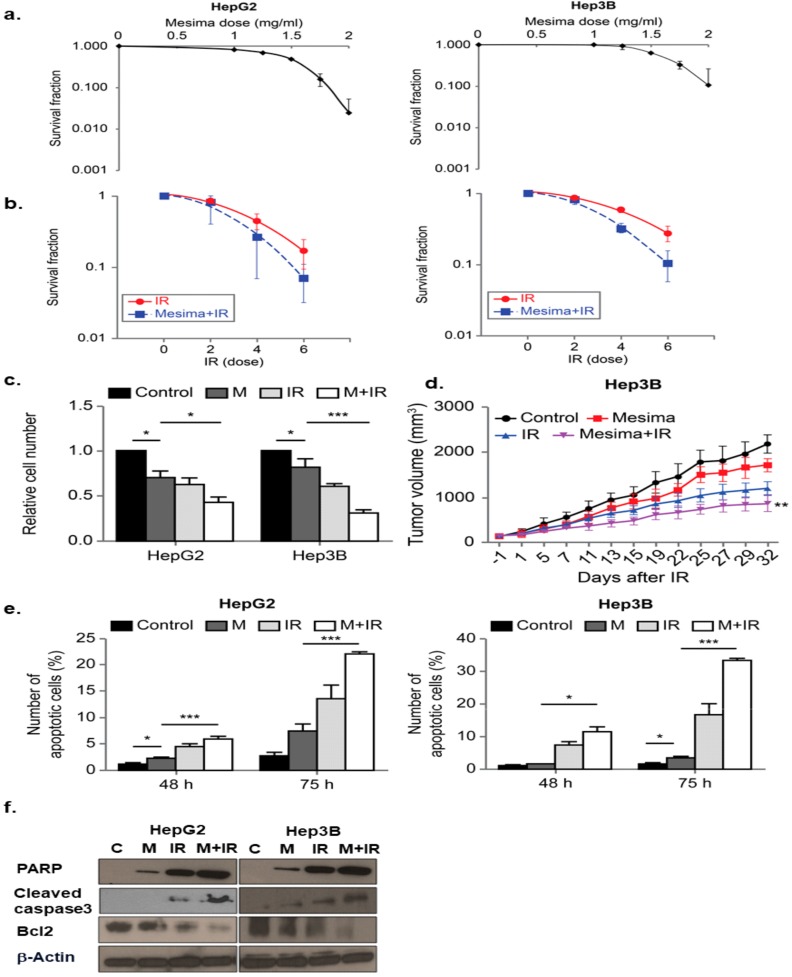
Radiosensitizing effects of mesima on hepatocellular carcinoma (HCC) cells. (**a**) Survival fraction of HepG2 and Hep3B cell lines treated with various concentrations of mesima for 72 h was measured by colony-forming assay. (**b**) Radiosensitivity of HepG2 and Hep3B cell lines with and without mesima (1.25 mg/mL) after various doses of γ-ray radiation was measured by colony-forming assay. Asterisks indicate values that are statistically significant in comparison with radiation only-treated cells. Values represent the means of three experiments ± SE; * *p* < 0.05; ** *p* < 0.01. (**c**) Cell viability was evaluated by cell counting using 0.4% trypan blue stain for HepG2 and Hep3B cells treated with mesima (1.25 mg/mL), irradiation (IR) (6 Gy), or in combination for the indicated durations; * *p* < 0.05; *** *p* < 0.001. (**d**) Nude mice were inoculated with Hep3B cells and treated with mesima, IR, or in combination; ** *p* < 0.01. (**e**) Two HCC cell lines were applied to a combination of mesima (1.25 mg/mL) and 6 Gy irradiation (IR). After 48 or 72 h, cells were subjected to annexin V and propidium iodide staining and analyzed by flow cytometry; * *p* < 0.05; *** *p* < 0.001. (**f**) Immunoblotting of cell lysates with the indicated antibodies.

We first used the clonogenic survival assay to determine the optimal mesima concentration to promote cancer cell death. Among various doses, 1.25 mg/mL of mesima was shown as the most effective combination with radiation in human HCC cell lines Hep3B and HepG2, relevant to an inhibitory concentration of 20% (Figure 1a). To decide the biological effects of mesima on radiation-induced toxicity, clonogenic survival was conducted. Figure 1b represents the dose-response curves of the two HCC cell lines irradiated with γ-ray beams in the presence of mesima. Survival fraction decreased in mesima-treated cells following γ-ray irradiation (IR) as compared with that of cells irradiated without mesima. The parameters of the linear quadratic fitting of survival curves and doses of IR required for 50% cell death with and without mesima were calculated from Figure 1b and are arranged in Table 1, Table 2, and Table 3. The effect of mesima on radiation-induced cell killing was showed as a radiosensitivity enhancement factor (REF) and dose reduction values (Table 4). Next, cell growth was checked by trypan blue cell viability after combination treatment on the two HCC cell lines (Figure 1c). Cell viability decreased in a combination treatment manner compared with that of the single treatment group. To study the biological effect of radiation plus mesima on HCC growth in vivo, we performed a subcutaneous HCC model made by injecting human Hep3B cells into mice. Figure 1d and Table 5 showed that groups treated with a combination of radiation and mesima showed decreased growth compared to that of the control group or single treating groups. Therefore, tumors in the single-treated groups were larger than those in the group treating combined treatment 

To study whether mesima and radiation enhanced the apoptosis, we performed early apoptosis by annexin V and PI staining [15]. In HCC cell lines, combination treatment considerably increased the portion of early apoptotic cells (Figure 1e). Flow cytometry and colony-forming assays provided correlated outcomes, revealing that greater sensitivity was afforded by mesima + radiation in comparison with either mesima or γ-ray irradiation treatment alone (Figure 1e). The cell viability rate reduced to a greater level for cells treated with mesima prior to IR compared with that of cells irradiated without mesima, showing that mesima works as a promising radiosensitizer to induce the radio-sensitivity of HCC cells to IR-induced cell killing, and that combined treatment likely controls the apoptotic process with intracellular caspase signaling. We next investigated whether the mesima-enhanced IR cytotoxicity resulted from further activation of caspase, a unique characteristic of apoptotic cell death [15], resulting in apoptotic cell death. We measured mesima + radiation-induced activation of caspase-3 protease activities. Moreover, caspase-3 activation led to cleavage of Poly [ADP-ribose] polymerase 1 (PARP-1), which can be detected via Western blotting by the appearance of a distinct band at 89 kDa, indicating cleaved PARP-1 [16]. The intensity of the cleaved PARP-1 band increased for up to 48 h after exposure to mesima + radiation (Figure 1f). These results indicated that the combined treatment likely regulated the apoptotic process, including intracellular caspase signaling.

### 2.2. Effects of Mesima and Radiation on the Cell Cycle

**Figure 2:**
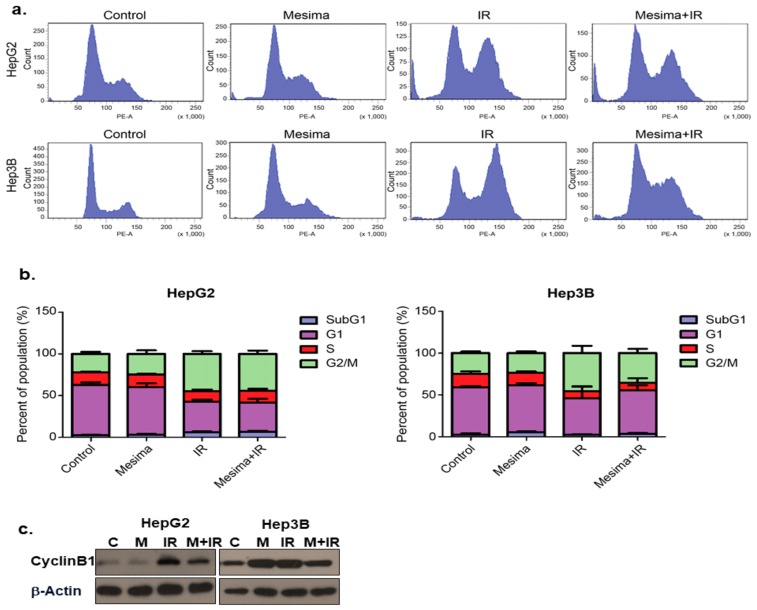
Mesima blocked cell cycle progression in the G2-M phase of irradiated cells and modulated the expression of cell cycle regulators. (**a**) Effects of mesima + radiation on cell cycle phase distribution. The two HCC cell lines were treated with a combination of mesima (1.25 mg/mL) and 6 Gy irradiation. After 24 h, the cell cycle distribution was analyzed quantitatively. Values represent the means of three experiments ± SE. (**b**) After 24 h, the cell cycle distribution was analyzed quantitatively. Values represent the means of three experiments ± SE. (**c**) Cyclin expression as analyzed by Western blotting. The two HCC cell lines were treated with radiation prior to mesima and were incubated for 24 h. Equal amounts of cell lysates (30 μg) were separated by electrophoresis and analyzed by Western blotting, as indicated.

We further studied whether mesima + radiation-induced toxicity resulted from changes in cell cycle regulation by defining cell cycle. Mesima + radiation treatment considerably reduced the portion of cells in G2 to M phase for Hep3B cells (but not HepG2 cells), compared to that from radiation alone at 24 h treatment (Figure 2a). The combination of mesima and IR markedly regulated cell cycle by blocking radiation-induced G2/M arrest in Hep3B, but not HepG2 cells (Figure 2b). In comparison, Western blotting revealed that radiation alone resulted in a significant accumulation of cyclin B, a key cell cycle regulator involved in the G2/M transition (Figure 2c), whereas combined metformin (an inducer of cell cycle arrest) and IR treatment reduced radiation-induced accumulation of cyclin B.

### 2.3. Effects of Mesima and Radiation on Autophagic Cell Death

**Figure 3:**
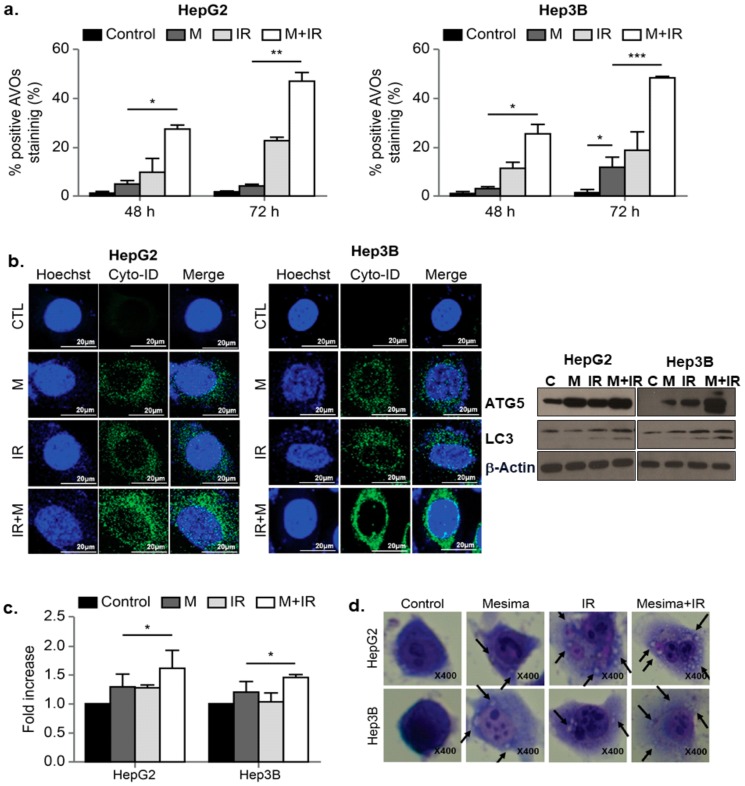
Effects of mesima and radiation on autophagy. (**a**) Development of acidic vesicular organelles (AVOs) in mesima and radiation-treated HepG2 and Hep3B cells. Green and red fluorescence in acridine orange (AO)-stained cells were detected using flow cytometry; * *p* < 0.05; ** *p* < 0.01; *** *p* < 0.001. (**b**) (left) Cyto-ID staining with and without mesima and radiation treatment in the two HCC cell lines; (right) immunoblotting of cell lysates with the indicated antibodies. (**c**) Mesima + radiation resulted in an increase in the CYTO-ID dye signal; * *p* < 0.05. (**d**) Cells were stained with Giemsa (10% in phosphate-buffered saline), washed, and imaged under a Leica DM IRB light microscope (magnification, 40×). Black arrows show vacuoles. A representative of two independent experiments is shown.

To study the anticancer effects of mesima and radiation, we performed another cellular response related to cell death upon mesima or radiation treatment; in particular, we defined the influences on autophagy, as mesima and radiation derive this phenomenon. Mesima-induced vacuole formation was assessed, and then we analyzed mesima plus IR-induced acidic vesicular organelle (AVO)-stained acidic vacuoles by flow cytometry. The level of AVOs revealed a time-dependent increase of AVOs in both HepG2 and Hep3B cells (Figure 3a). As shown in Figure 3b, increase of Cyto-ID Green, an autophagy indicator, was shown in combination-treated HepG2 and Hep3B cells. Western blot analyses revealed that LC3, a specific marker of autophagosome promotion, was increased in combination treatment compared with that of single treatment; furthermore, mesima + IR treatment increased Autophagy related 5 (Atg5) levels compared with that of single treatment (Figure 3b, right). Autophagy was quantified using the green Cyto-ID dye, which specifically fluoresces in autophagic vesicles (Figure 3c). The Cyto-ID dye accumulated in mesima + IR-treated HCC cells in comparison with those treated with radiation alone. Notably, these data are correlated with the morphological changes observed through Giemsa staining (Figure 3d). Together, our data showed that mesima + radiation treatment increased the numbers of both autophagic markers (vacuoles) and dead cells.

### 2.4. Effects of Mesima and Radiation on DNA Damage

**Figure 4:**
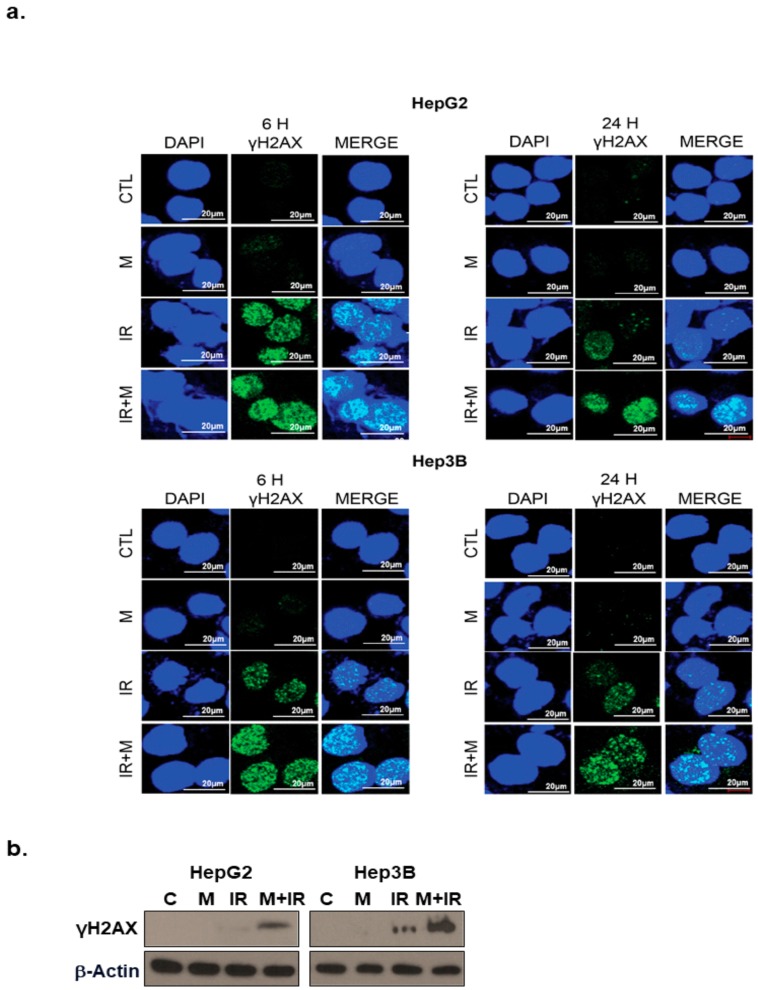
Effects of mesima on the DNA damage response in irradiated HCC cells. (**a**) Immunocytochemistry for phosphorylated H2AX, a marker of the DNA damage response, in HCC cells treated to radiation in the presence of mesima at 6 and 24 h after irradiation. (**b**) Immunoblotting of cell lysates with the indicated antibodies. CTL: Control.

To investigate whether mesima altered the repair of double-strand breaks (DSBs) induced by IR treatment, we checked the pattern of γ-H2AX foci, a marker for DSBs, in cells treated with mesima and/or IR. Our finding showed that an increase of γ-H2AX foci happened 24 h after radiation treatment in the existence of mesima (Figure 4a,b). Furthermore, mesima + radiation treatment postponed the clearance of γ-H2AX (Figure 4a), suggesting that mesima maintains IR-induced DNA damage and therefore enhances the radiosensitivity of cells.

### 2.5. Combination Therapy Significantly Inhibited Tumor Cell Motility and Invasion

**Figure 5:**
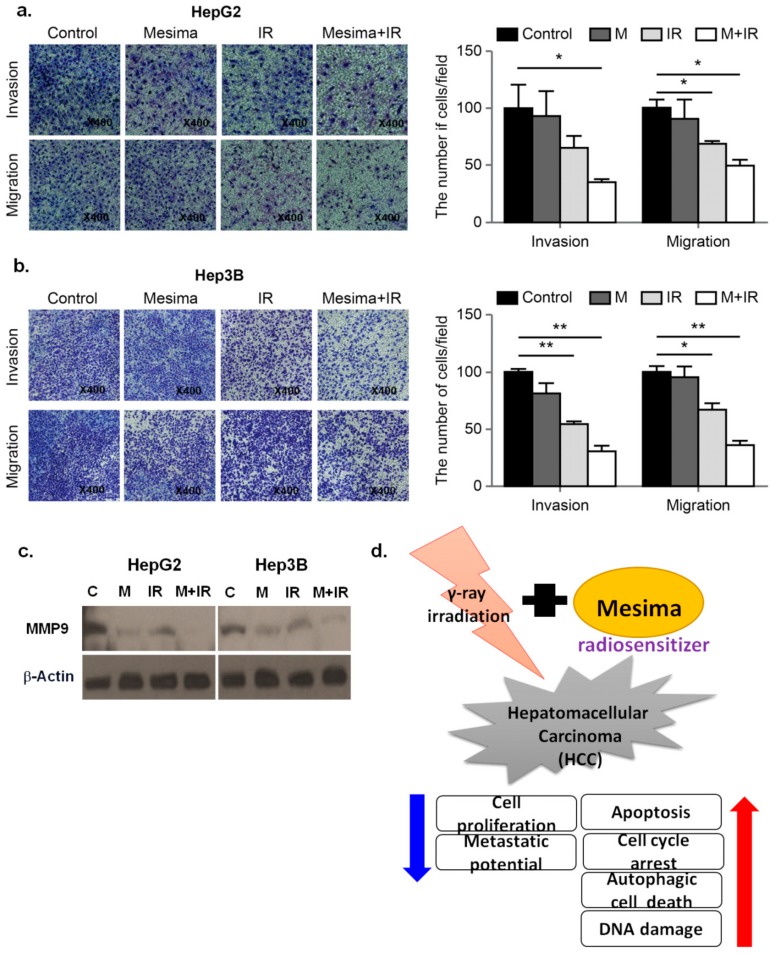
Effect of treatment with mesima and radiation on the invasion and migration of HCC cells. (**a**,**b**) HCC cell invasion and migration after 24 h mesima + radiation treatment were conducted by Transwell chamber assays; * *p* < 0.05; ** *p* < 0.01. (**c**) Cell lysates (30 μg) of HCC cells were immunoblotted with MMP-9. (**d**) The proposed mechanisms for mesima as a radiosensitizer of HCC cells.

Cell migration and invasion played critical functions in tumor metastasis potentials [17]. To study the anti-metastatic effect of mesima, the migration and invasion abilities of HCC cells following mesima and mesima + radiation treatment were performed by Transwell assays. As shown in Figure 5a,b, mesima + radiation considerably decreased the migration and invasion of the two HCC cell lines in Transwell chamber assays. Moreover, we evaluated the extent to which Metrix metallopeptidase 9 (MMP-9 ) expression was altered in HCC cells treated with mesima + radiation. As predicted, mesima + IR down-regulated MMP-9, which promoted invasion in HCC cells (Figure 5c). The proposed mechanisms for mesima as a radiosensitizer of HCC cells are shown in Figure 5d.

Moreover, we evaluated the extent to which MMP-9 expression was altered in HCC cells treated with mesima + radiation. As predicted, mesima + IR down-regulated MMP-9, which promoted invasion in HCC cells.

## 3. Discussion

Recently, the need to advance anticancer drugs such as tumor-specific radiosensitizers to finally increase the therapeutic ratio and block tumor radioresistance has become apparent. In addition, the utilization of natural products (NPs) as antitumor agents for the control of human cancers has been considered a good choice because they are available for use and often show little or no toxicity. Accordingly, because of high cost, low level of side effects, and treatment limitations of conventional chemotherapy and radiotherapy, it is calculated that about 40% of Americans use alternative drugs including herbal medicine and NPs for cancer treatment [18]. In our study, we provided, to our knowledge, the first piece of evidence that the NP mesima, which has been shown to have both anticancer and chemopreventive potential, also shows radiosensitization ability for HCC in vitro and in vivo.

The biologic effects of mesima are varied [11,12,13,14] and its use has become popular as a complementary treatment for cancers in recent years. To study its mechanism in HCC, a clonogenic survival assay and in vivo xenograft model of mesima-treated HCC cancer cells were performed. We then confirmed the radiosensitizing effect of mesima with a clonogenic assay, which was also selected to decide cell reproductive death after treatment with IR [19]. It was shown that through pretreatment with mesima before radiation exposure that the surviving fraction reduced significantly (Figure 1). In two HCC cell lines, 48 h exposure to mesima + radiation treatment considerably increased the portion of early apoptotic cells (Figure 1). Together, these data showed that mesima exhibits the activity for radiosensitization of HCC cells.

Various clinical trials have begun to prove the utilization of mesima combined with several anticancer drugs to treat many cancers. We therefore confirmed whether mesima was also able to increase the inhibitory effects of radiation on cell cycle as a critical point in regulating radiosensitivity. Cells show different radiosensitivity in various stages of the cell cycle, with the G2/M phase being the most sensitive to IR [20]. In the present study, the cell portion of the G2/M phase upon combination treatment showed a decrease compared to that following single treatment (Figure 2). Mesima also increased the autophagy induced by radiation, as indicated by increased AVO staining, expression of Cyto-ID Green as an autophagy mark, and levels of LC3, a specific indicator of autophagosome formation. Furthermore, Giemsa-stained combination-treated HCC cells sowed structural changes in the cytoplasm and membranes compared to those of single treatment (Figure 3).

IR treatment also affects DNA damage such as DSBs, thereby it can be clue for development of various treatment in tumor cells [21]. The evidence for cytotoxic chemotherapy as a radiosensitizer is based on the theory that further DNA damage would decrease the threshold of cell death induced by IR. Therefore, to study whether mesima affected DSB repair, we checked the events of γ-H2AX foci in cells treated with mesima and/or IR. Our results found that mesima both enhanced and prolonged DNA damage and therefore enhanced the radiosensitivity of cells (Figure 4). Next, we needed to define how mesima decreased the repair activity of IR-induced DSBs and the effects of mesima involved in the Non-homologous end joining (NHEJ) and Homologous recombination (HR) pathways, showing the block of the DNA DSB repair pathway in HCC cells.

Finally, we further investigated the metastatic potentials of mesima + radiation treatment (Figure 5). To find the proteins related to the inhibition of invasion mediated by mesima and IR, we studied the expression of proteins related to the epithelial to mesenchymal transition through Western blot analysis and Transwell chamber assay. The expression of MMP-9 was shown to increase metastatic potentials of cancer cells. The functions of MMP-9 are the regulation of cancer progression, angiogenesis, and recruitment of macrophages or other bone marrow-derived myeloid cells to the existing metastatic niche [22]. Further, mesima treatment alone inhibited the invasive and migratory abilities of HCC cells, with its effect being synergistically assessed upon mesima + radiation combination treatment. Therefore, the combined results indicated that the effects of mesina with regard to regulating radiosensitization were mainly related to apoptosis, cell cycle, autophagy, DNA repair, and metastatic potential, thus highlighting it as a better agent to study as a radiosensitizer.

## 4. Materials and Methods

### 4.1. Cell Culture

Human hepatocarcinoma Hep3B cancer cells were cultured in Dulbecco’s minimal essential medium (GIBCO, Gaithersburg, MD, USA) supplemented with heat-inactivated 10% fetal bovine serum (FBS; GIBCO), 0.1 mM non-essential amino acids, glutamine, 4-(2-hydroxyethyl)-1-piperazineethanesulfonic acid (HEPES), and antibiotics at 37 °C in a 5% CO_2_-humidified incubator. Human hepatocarcinoma HepG2 cells were grown in Roswell Park Memorial Institute (RPMI) 1640 medium supplemented with 10% FBS, glutamine, HEPES, and antibiotics at 37 °C in a 5% CO_2_ humidified incubator.

### 4.2. Irradiation

Cells were plated in dishes and incubated at 37 °C and 5% CO_2_ under humidified conditions to 70–80% confluence. Cells were irradiated using a ^137^Cesium γ-ray source (Atomic Energy of Canada, Ontario, Canada) at a dose rate of 3.81 Gy/min.

### 4.3. Western Blotting

Mesima were used to treat the cells for 24 h and then incubated for the indicated time after irradiation. The cells were then lysed with Cell Lysis buffer (Cell Signaling Technology). Proteins (30 μg) were separated by sodium-polyacrylamide gel electrophoresis and transferred to nitrocellulose membranes. The membranes were blocked with 1% (*v*/*v*) non-fat dried milk in Tris-buffered saline with 0.05% Tween-20 and incubated with the indicated antibodies. Primary antibodies were used at a 1:1000 dilution, and secondary antibodies were used at a 1:5000 dilution. Immunoreactive protein bands were visualized by enhanced chemiluminescence (Thermo Fisher Scientific, Waltham, MA, USA) and scanned. Data of Western blot were independently repeated 2 or 3 times.

### 4.4. Antibodies and Chemicals

Anti-PARP, cleaved-caspase3, Bcl2, CyclinB1, γH2AX, ATG5, LC3(A/B), and MMP-9 were purchased from Cell Signaling Technology (Danvers, MA, USA) and anti-β-actin was purchased from Santa Cruz Biotechnology (Dallas, TX, USA). Mesima was obtained from Han Kook Shin Yak Pharmaceutical Co., Ltd.

### 4.5. Colony-Forming Assay

Cells (500–1000) were seeded into 60 mm dishes in triplicate and maintained at 1.25 mg/mL mesima for 24 h prior to IR. After 8–10 days, colonies were fixed with methanol and stained with 0.4% crystal violet (Sigma, St. Louis, MO, USA).

### 4.6. Xenograft Tumors in Nude Mice

Four-week-old specific pathogen-free male Balb/c nude mice (Orientbio Inc., Gapyeong, Korea) maintained in a laminar air flow cabinet at a regulated temperature (22 ± 3 °C) and humidity (approximately 30 ± 20%), were housed under a 12 h/12 h light/dark cycle, and fed ad libitum with rodent diet and water. Hep3B xenograft mice were generated by subcutaneous injection of 3 × 10^6^ Hep3B cells into the right thigh. When the tumor reached a volume of approximately 150 mm^3^, the mice were randomly divided into four groups (*n* = 8): control, mesima alone, radiation, and mesima + radiation. From grouping to euthanasia, mesima was orally administered daily at a concentration of 100 mg/kg. When the tumor volume of the control group attained about 200 mm^3^, the radiation groups (radiation alone and mesima + radaition) were treated with a single 6 Gy fraction of local-regional irradiation using a ^60^Co γ-ray irradiator (Theratron 780, Atomic Energy of Canada, Chalk River, Ontario, Canada). The tumor volume (V) was calculated using the standard formula: V(mm^3^) = π/6 × (smaller diameter)^2^ × (larger diameter). Mice were euthanized by carbon dioxide (CO_2_) inhalation when the mean tumor volume of the control group was 2000 mm^3^. All animal protocols and studies were approved by the Institutional Animal Care and use Committee (IACUC) of the Korean Institute of Radiological and Medical Sciences (KIRAMS 2017-0090).

### 4.7. Detection of Apoptotic Cells by Annexin V Staining

Following exposure to 1.25 mg/mL mesima for 24 h mesima, IR was applied to the cells, which were then incubated for a further 48 h. Cells were washed with ice-cold Phosphate-buffered saline (PBS), trypsinized, and re-suspended in 1X binding buffer (10 mM HEPES/NaOH (pH 7.4), 140 mM NaCl, and 2.5 mM CaCl_2_) at 1 × 10^6^ cells/mL.

### 4.8. Analysis of Cell Cycle Progression

Cells were seeded in 60 mm dishes at 60% confluency. After an indicated time point, cells were trypsinized, harvested, and fixed in 1 mL of 70% cold ethanol in test tubes and incubated at 4 °C overnight. After incubation, cells were centrifuged at 2000 rpm for 3 min, and the cell pellets were resuspended in 500 μL propidium iodine (10 μg/mL) containing 300 μg/ml RNase (Sigma). Cell cycle distribution was analyzed from 10,000 cells with CellQuest software using FACScaliber.

### 4.9. Quantification of AVOs by Acridine Orange (AO) Staining

Cells were treated with mesima for the indicated time points, followed by staining with 1 μM AO for 15 min. Cells were then washed, re-suspended in PBS, and subjected to FACS analysis. The green (510–530 nm, FL-1) and red (650 nm, FL-3) fluorescence of AO, following blue (488 nm) excitation, was determined over 10,000 events and measured on a FACScan cytofluorimeter using Cell Quest software.

### 4.10. Immunofluorescence

Immunocytochemistry was conducted to define the nuclear distribution of γ-H2AX in individual cells. Cells were grown on chambered slides for 1 day prior to IR and/or mesima treatment. After 24 h mesima exposure, cells were irradiated and treated for 6 or 24 h. All treatments were performed while cells remained attached to the slides, followed by reported as previously [23].

### 4.11. Morphology

To examine the effect of mesima + IR on cell morphology, treated cells were fixed with methanol for 10 min and then stained with Giemsa (10% in PBS) for 15 min, followed by washing with tap water. Images were acquired using a Nikon Eclipse Ts2R-FL microscope (Tokyo, Japan).

### 4.12. Transwell Chamber Assay

The invasive ability of HCC cells was measured using Transwell chambers according to the manufacturer’s protocol [23]. Cells were seeded onto the membrane of the upper chamber of the Transwell at a concentration of 4 × 10^5^ cells/mL in 150 μL of medium, and were left untreated or treated with the indicated doses of mesima, IR, or mesima + IR for 24 h. The medium in the upper chamber was serum-free, whereas the medium in the lower chamber contained 10% (*v/v*) FBS as a source of chemo-attractants. Cells that passed through the Matrigel/gelatin-coated membrane were stained with Cell Stain Solution containing crystal violet supplied in the Transwell chamber assay (Chemicon, Millipore, Billerica, MA, USA) and photographed after a 24 h incubation period.

### 4.13. Statistical Analysis

Statistical significance was determined by Student’s *t*-test. Differences were considered significant if the *p*-value was <0.05, 0.01, or 0.001.

## 5. Conclusions

In summary, we showed that mesima induced the therapeutic efficacy of radiation by decreasing cancer cell survival, apoptosis, cell cycle regulation, DNA repair, autophagy, and cancer cell metastatic potentials. These data suggest molecular clues for the utilization of chemo-radiation to treat HCC cancer via an in vivo model. Accordingly, we provided the scientific evidence for mesima as a radiosensitizer in HCC.

## Figures and Tables

**Table 1:** Linear quadratic fitting parameters α and β for survival curves in cells irradiated in the presence or absence of mesima and incubated for 14 days.

Cell Type	Treatment	α (Gy^-1^)	β (Gy^-1^)
HepG2	γ-ray	0.249 ± 0.756	0.172 ± 0.219
	γ-ray + mesima	0.328 ± 0.777	0.248 ± 0.226
Hep3B	γ-ray	0.185 ± 0.748	0.124 ± 0.216
	γ-ray + mesima	0.265 ± 0.767	0.206 ± 0.223

**Table 2:** Radiation dose needed to kill 50% of cells (D50) in the presence or absence of mesima. Values were obtained from Figure 1a.

Cell Type	Radiation	D50 without Mesima	D50 with Mesima
HepG2	γ-ray	1.42 Gy	1.14 Gy
Hep3B	γ-ray	1.73 Gy	1.30 Gy

**Table 3:** The plating efficiencies for controls with and without mesima.

Cell Type	With Mesima (%)	Without Mesima (%)
HepG2	35 ± 11	62 ± 10
Hep3B	63 ± 3	82 ± 9

**Table 4:** Radiosensitivity enhancement factor (REF) and dose reduction values.

Cell Type	Radiation	REF Value	Dose Reduction (%)
HepG2	γ-ray	1.24 Gy	80.3
Hep3B	γ-ray	1.33 Gy	75.1

REF: ratio of the radiation dose required to kill 50% of cells in the presence of mesima divided by the dose in the absence of mesima.

**Table 5:** Tumor growth inhibition (TGI) compared to control group or radiation group in the presence or absence of mesima. Values were obtained from Figure 1d.

Vs. Control	% of TGI	Vs. Radiation	% of TGI
Mesima	21.6	Mesima + radiation	28.3
Radiation	44.9		
Mesima + radiation	60.5		

## References

[1] Parkin D.M. (2001). Global cancer statistics in the year 2000. Lancet Oncol..

[2] El–Serag H.B., Rudolph K.L. (2007). Hepatocellular carcinoma: Epidemiology and molecular carcinogenesis. Gastroenterology.

[3] Bruix J., Sherman M.J.H. (2005). Management of hepatocellular carcinoma. Hepatology.

[4] Sliva D. (2010). Medicinal mushroom Phellinus linteus as an alternative cancer therapy. Exp. Ther. Med..

[5] Song K.-S., Cho S.-M., Lee J., Kim H.-M., Han S.-B., Ko K.-S., Yoo I.-D. (1995). B-lymphocyte-stimulating polysaccharide from mushroom Phellinus linteus. Chem. Pharm. Bull..

[6] Han S.B., Lee C.W., Jeon Y.J., Hong N.D., Yoo I.D., Yang K.-H., Kim H.M. (1999). The inhibitory effect of polysaccharides isolated from Phellinus linteus on tumor growth and metastasis. Immunopharmacology.

[7] Borchers A.T., Stern J.S., Hackman R.M., Keen C.L., Gershwin M.E. (1999). Medicine, Mushrooms, tumors, and immunity. Proc. Soc. Exp. Biol. Med..

[8] Chihara G., Maeda Y., Hamuro J., Sasaki T., Fukuoka F. (1969). Inhibition of mouse sarcoma 180 by polysaccharides from Lentinus edodes (Berk.) sing. Nature.

[9] Wasser S.P. (2002). Biotechnology, Medicinal mushrooms as a source of antitumor and immunomodulating polysaccharides. Appl. Microbiol. Biotechnol..

[10] Guo J., Zhu T., Collins L., Xiao Z.X.J., Kim S.H., Chen C.Y. (2007). Modulation of lung cancer growth arrest and apoptosis by Phellinus Linteus. Mol. Carcinog..

[11] Collins L., Zhu T., Guo J., Xiao Z., Chen C.Y. (2006). Phellinus linteus sensitises apoptosis induced by doxorubicin in prostate cancer. Br. J. Cancer.

[12] Song K.-S., Li G., Kim J.-S., Jing K., Kim T.-D., Kim J.-P., Seo S.-B., Yoo J.-K., Park H.-D., Hwang B.-D. (2011). Protein-bound polysaccharide from Phellinus linteus inhibits tumor growth, invasion, and angiogenesis and alters Wnt/β-catenin in SW480 human colon cancer cells. BMC Cancer.

[13] Li G., Kim D.-H., Kim T.-D., Park B.-J., Park H.-D., Park J.-I., Na M.-K., Kim H.-C., Hong N.-D., Lim K. (2004). Protein-bound polysaccharide from Phellinus linteus induces G2/M phase arrest and apoptosis in SW480 human colon cancer cells. Cancer Lett..

[14] Lee H.-J., Lee H.-J., Lim E.-S., Ahn K.-S., Shim B.-S., Kim H.-M., Gong S.-J., Kim D.-K., Kim S.-H. (2005). Cambodian Phellinus linteus inhibits experimental metastasis of melanoma cells in mice via regulation of urokinase type plasminogen activator. Biol. Pharm. Bull..

[15] McIlwain D.R., Berger T., Mak T.W. (2013). Caspase functions in cell death and disease. Cold Spring Harb. Perspect. Biol..

[16] Los M., Mozoluk M., Ferrari D., Stepczynska A., Stroh C., Renz A., Herceg Z., Wang Z.-Q., Schulze-Osthoff K. (2002). Activation and caspase-mediated inhibition of PARP: A molecular switch between fibroblast necrosis and apoptosis in death receptor signaling. Mol. Biol. Cell.

[17] Leber M.F., Efferth T. (2009). Molecular principles of cancer invasion and metastasis. Int. J. Oncol..

[18] Gullett N.P., Amin A.R., Bayraktar S., Pezzuto J.M., Shin D.M., Khuri F.R., Aggarwal B.B., Surh Y.-J., Kucuk O. (2010). Cancer prevention with natural compounds. Semin. Oncol..

[19] Franken N.A., Rodermond H.M., Stap J., Haveman J., Van Bree C.J.N. (2006). Clonogenic assay of cells in vitro. Nature Protoc..

[20] Pawlik T.M., Keyomarsi K. (2004). Role of cell cycle in mediating sensitivity to radiotherapy. Int. J. Radiat. Oncol. Biol. Phys..

[21] Jackson S.P. (2002). Sensing and repairing DNA double-strand breaks. Carcinogenesis.

[22] Psaila B., Lyden D. (2009). The metastatic niche: Adapting the foreign soil. Nat. Rev. Cancer.

[23] Kim E.H., Song H.S., Yoo S.H., Yoon M. (2016). Tumor treating fields inhibit glioblastoma cell migration, invasion and angiogenesis. Oncotarget.

